# TROP2 Expressed in the Trunk of the Ureteric Duct Regulates Branching Morphogenesis during Kidney Development

**DOI:** 10.1371/journal.pone.0028607

**Published:** 2011-12-14

**Authors:** Yuko Tsukahara, Minoru Tanaka, Atsushi Miyajima

**Affiliations:** Institute of Molecular and Cellular Biosciences, The University of Tokyo, Tokyo, Japan; Feinberg Cardiovascular Research Institute, Northwestern University, United States of America

## Abstract

TROP2, a cell surface protein structurally related to EpCAM, is expressed in various carcinomas, though its function remains largely unknown. We examined the expression of TROP2 and EpCAM in fetal mouse tissues, and found distinct patterns in the ureteric bud of the fetal kidney, which forms a tree-like structure. The tip cells in the ureteric bud proliferate to form branches, whereas the trunk cells differentiate to form a polarized ductal structure. EpCAM was expressed throughout the ureteric bud, whereas TROP2 expression was strongest at the trunk but diminished towards the tips, indicating the distinct cell populations in the ureteric bud. The cells highly expressing TROP2 (TROP2^high^) were negative for Ki67, a proliferating cell marker, and TROP2 and collagen-I were co-localized to the basal membrane of the trunk cells. TROP2^high^ cells isolated from the fetal kidney failed to attach and spread on collagen-coated plates. Using MDCK cells, a well-established model for studying the branching morphogenesis of the ureteric bud, TROP2 was shown to inhibit cell spreading and motility on collagen-coated plates, and also branching in collagen-gel cultures, which mimic the ureteric bud's microenvironment. These results together suggest that TROP2 modulates the interaction between the cells and matrix and regulates the formation of the ureteric duct by suppressing branching from the trunk during kidney development.

## Introduction

TROP2, also known as EGP-1, M1S1, GA733-1, and TACSTD2, is a 36-kDa membrane glycoprotein, initially identified as a tumor-associated calcium signal transducer (TACSTD) expressed in gastrointestinal, bladder, lung, and cervix carcinomas and later found to be highly expressed in many other tumors including pancreatic cancers and squamous cell carcinomas of the oral cavity [1]. TROP2 is strongly expressed in trophoblasts forming the outer layer of the blastocyst which develops into the placenta [2]. In the prostate, TROP2 is specifically expressed in prostate stem cells located in the basal region [3]. We have recently found that TROP2 is expressed in hepatic progenitor cells known as oval cells, which are produced in response to severe liver injury and form duct-like structures surrounding the portal vein [4].

TROP2 is structurally related to epithelial cell adhesion molecule (EpCAM), also known as TACSTD1. Both TROP2 and EpCAM are type I transmembrane proteins, consisting of a hydrophobic leader peptide, a thyroglobulin-like domain, a single transmembrane domain and a short cytoplasmic domain. The cytoplasmic tail of TROP2, but not EpCAM, contains a region homologous to phosphatidylinositol 4,5-bisphosphate (PIP2)-binding motif [5]. EpCAM is widely expressed in epithelial cells and plays roles in cell-cell adhesion, cell proliferation and cell motility [6]–[10]. On the other hand, TROP2 was suggested to enhance the proliferation of tumor cells in an anchorage-independent manner [11] and the formation of tight junctions by interacting with Claudin-1 and -7 in the corneal epithelium [12]. However, TROP2 expression in normal tissues and its functions still remain largely unstudied.

In the development of the mammalian kidney, the ureteric bud epithelium proliferates and branches to form a tree-like structure [13], [14]. This process is known as branching morphogenesis and depends on interactions of the ureteric bud with the metanephric mesenchyme and also components of the extracellular matrix (ECM). Factors secreted from the metanephric mesenchyme enhance the proliferation, elongation and branching of the ureteric bud cells, resulting in differentiation to form the collecting duct system. On the other hand, ECM components around the ureteric bud modulate growth factor signaling and provide an anchorage for cell migration via the ECM-specific integrin receptors on the ureteric bud cells [15], [16].

The cells forming the tip and trunk of the ureteric bud exhibit different characteristics [17]. The tip cells are proliferating immature cells that are located at the branching points and interact with the metanephric mesenchyme. By contrast, the trunk cells are differentiating into the collecting duct with a rigid structure consisting of polarized epithelial cells. While the molecular mechanisms underlying the establishment of the tip structure have been well studied, little is known about the mechanisms of trunk morphogenesis. In particular, the factors linking trunk cells to the extracellular environment remain totally unknown.

To gain insights into the functions of TROP2, we examined the expression of TROP2 together with EpCAM during mouse development and found that the two differ significantly. During development, EpCAM was widely expressed in epithelial tissues, whereas TROP2 expression was restricted to EpCAM^+^ cells in a few organs. In particular, a unique pattern of TROP2 expression was found in fetal kidney, i.e., TROP2 expression increases from the tip toward the trunk of the ureteric bud, whereas EpCAM was expressed uniformly. In this study, we show that the expression pattern does not appear to correlate with the known functions of TROP2 in cell proliferation and tight junction formation. Using anti-TROP2 and anti-EpCAM antibodies (Abs), we isolated the tip cells and trunk cells of the ureteric buds, and revealed that their morphology on collagen-gel differs significantly, i.e. tip cells spread, whereas trunk cells remain a rounded shape. We further demonstrate that TROP2 alters cell shape and motility on collagen-gel using a primary culture of ureteric bud cells and that it also inhibits branching of MDCK cells. Our findings suggest that TROP2 plays a role in the development of a ductal structure of ureteric trunks by suppressing the formation of new branches.

## Results

### Expression of TROP2 during kidney development

**Figure 1:**
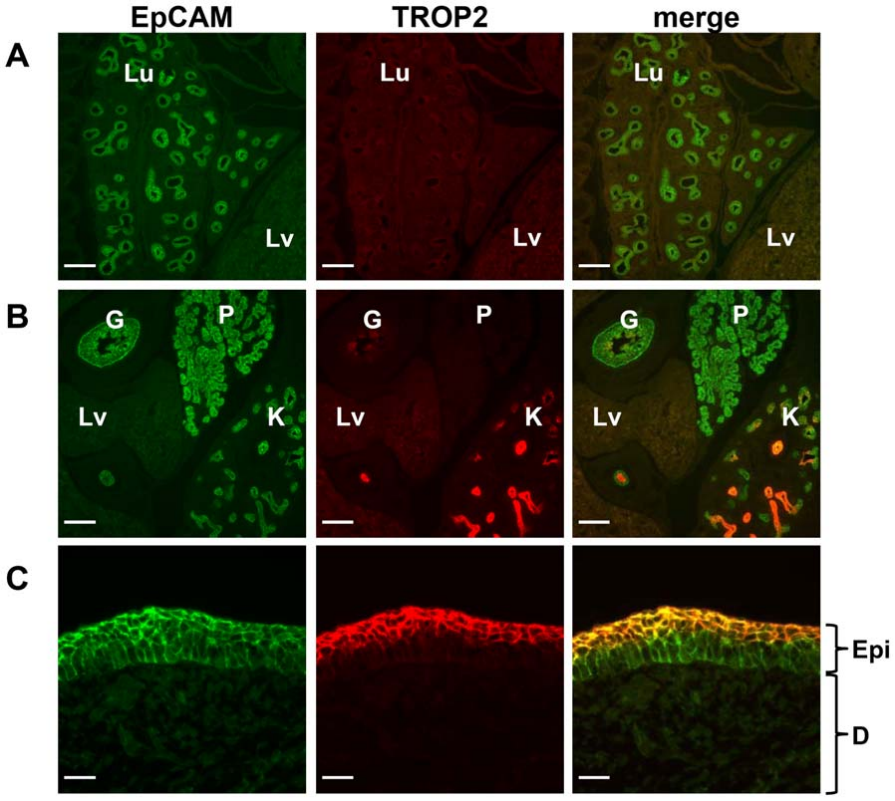
Expression of TROP2 and EpCAM in mouse at E14.5. (A–C) Immunostaining of E14.5 mouse frozen sections with anti-EpCAM (green) and anti-TROP2 (red) Abs. EpCAM was expressed in epithelial cells of various organs. By contrast, TROP2 expression was restricted to EpCAM^+^ cells in the kidney, gut and epidermis. L ; Lung, Lv ; Liver, P ; Pancreas, G ; Gut, K ; Kidney, Epi ; Epidermis, D ; Dermis, Scale bars = 200 µm.

We performed an immunohistochemical analysis of mouse embryos by using anti-EpCAM and anti-TROP2 Abs. At embryonic day 14.5 (E14.5), EpCAM was expressed in a variety of epithelial tissues including lung, gut, kidney, pancreas and epidermis. By contrast, TROP2 expression was restricted to a few tissues including kidney, gut and epidermis, and the TROP2^+^ cells were a subpopulation of EpCAM^+^ cells (Fig. 1A–C). Because TROP2 was highly expressed in the kidney, we focused on the expression and role of TROP2 in kidney development.

**Figure 2:**
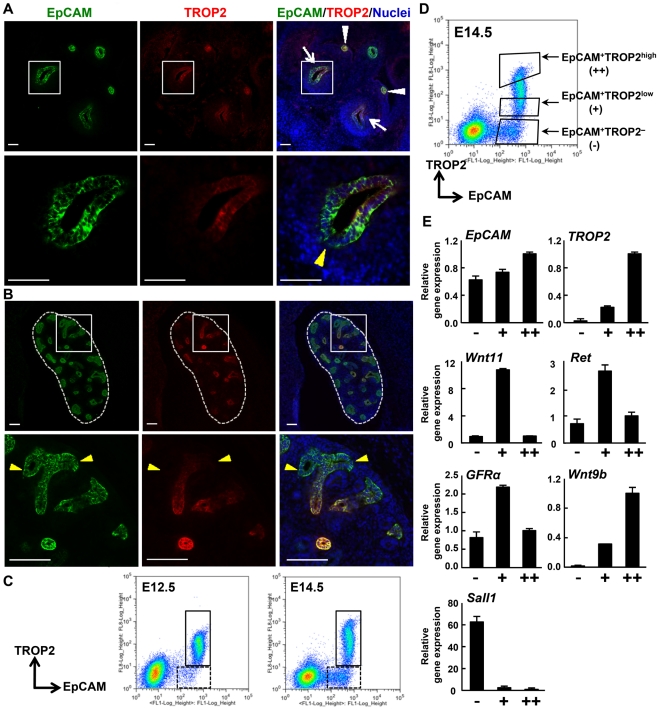
Characteristics of ureteric bud cells in the developing kidney. (A–B) Immunostaining of E11.5 (A) and E14.5 (B) kidney with anti-EpCAM (green) and anti-TROP2 (red) Abs and nuclei (blue). (A) Arrowheads indicate the Wolffian duct and the arrow indicates the ureteric bud. Higher magnification images of the boxed regions are shown underneath. In the lower figure of A, the ureteric bud grows toward the bottom and the yellow arrowhead indicates the tip of the ureteric bud. Scale bars = 50 µm. (B) The dotted line delineates the periphery of the kidney. Scale bars = 100 µm. (C) Flow cytometry of E12.5 and E14.5 kidney cells using anti-EpCAM and anti-TROP2 Abs. The boxed regions and the dotted boxed regions show TROP2^+^ and TROP2^−^ cell populations, respectively. (D) Fractionation of EpCAM+ cells in E14.5 kidney using anti-EpCAM and anti-TROP2 Abs. The upper, middle and lower boxed regions in the panel indicate the EpCAM^+^TROP2^high^, EpCAM^+^TROP2^low^ and EpCAM^+^TROP2^nega^ cell population, respectively. (E) Quantitative RT-PCR analysis of marker genes in freshly isolated E14.5 EpCAM^+^TROP2^nega^(−), EpCAM^+^TROP2^low^(+) and EpCAM^+^TROP2^high^(++) cells. The relative gene expression of marker/GAPDH is shown. Error bars are s.d.

In mice, the ureteric bud emerges at E10.5 from the Wolffian duct, which is induced by GDNF from metanephric mesenchyme [18], [19]. Subsequently, the ureteric buds are also induced to form the first branch at E11.5 and undergo further branching until E14.5∼15.0 by the signals from metanephric mesenchyme. Immunostaining in the fetal kidney at E11.5 revealed that EpCAM was uniformly expressed in the ureteric bud and the Wolffian duct (Figure 2A, arrow and arrowhead, respectively) [20]. Interestingly, TROP2 was expressed at the trunk of the ureteric bud and the Wolffian duct, but its expression was weak at the tip of the ureteric bud that is surrounded by metanephric mesenchyme (Figure 2A, yellow arrowhead). At E14.5, although EpCAM was expressed uniformly throughout the ureteric buds, TROP2 was expressed at the trunk but barely detectable at the tip (Figure 2B, yellow arrowhead). This gradient of TROP2 expression at the ureteric bud suggests TROP2 to be involved in morphogenesis of the ureteric bud, especially in the trunk.

### Characterization of TROP2^+^ and EpCAM^+^ cells in fetal kidney

To characterize the cells expressing TROP2 in the ureteric bud, we performed a flow cytometric analysis and revealed that EpCAM**^+^** cells in E12.5 kidney were divided into at least 2 populations, TROP2**^+^** and TROP2^−^ cells (Figure 2C). At E14.5, TROP2 expression was up-regulated, resulting in a broad distribution of TROP2 expression from TROP2^low^ to TROP2^high^. In addition, the EpCAM**^+^**TROP2^−^ cells increased during development, which is consistent with the results of immunostaining. These results suggested that the expression profiles of EpCAM and TROP2 define the distinct cell types in the developing urinary duct. To characterize E14.5 kidney cells, EpCAM^+^ cells were fractionated into three populations by cell sorting, EpCAM**^+^**TROP2^−^, EpCAM**^+^**TROP2^low^ and EpCAM**^+^**TROP2^high^ cells, based on the expression levels of TROP2 (Figure 2D).

The tip and trunk cells of the ureteric bud can be distinguished by the expression of specific markers. Quantitative RT-PCR revealed that Ret, Wnt11 and GFRα, tip cell markers [21]–[24], were highly expressed in EpCAM**^+^**TROP2^low^ cells, whereas Wnt9b, a trunk cell marker [25], was strongly expressed in EpCAM**^+^**TROP2^high^ cells (Figure 2E). These results indicate that the EpCAM**^+^**TROP2^low^ and EpCAM**^+^**TROP2^high^ populations include the tip and trunk cells, respectively, and that ureteric bud cells at different locations can be isolated by cell sorting based on TROP2 expression. It has been reported that EpCAM is expressed in the ureteric bud and also in the tissue derived from the metanephric mesenchyme by the mesenchymal-epithelial transition (MET) [20]. We found that EpCAM**^+^**TROP2^−^ cells expressed Sall1, a marker of metanephric mesenchymal cells [26](Figure 2E), and that the number of EpCAM**^+^**TROP2^−^ cells increased from E12.5 to E14.5, corresponding to the timing of MET [27](Figure 2C). These results suggest that the EpCAM**^+^**TROP2^−^ population is mainly composed of epithelial cells derived from the metanephric mesenchyme.

### Expression of TROP2 in ureteric buds

TROP2 is highly expressed in a variety of carcinomas and involved in cancer cell proliferation [11], [28]. In kidney development, a previous study showed that cell proliferation is restricted mainly to the tip of the ureteric bud in fetal kidney, which corresponds to TROP2^low^ cells [17]. In fact, immunostaining of Ki67, a marker of cell proliferation, showed the expression to be higher in the tip cells than in the trunk cells (Figure S1). This result suggests that, unlike in carcinomas, the TROP2 expression level is not positively correlated with cell proliferation in fetal kidney.

Furthermore, in the corneal epithelia, TROP2 enhances the expression and localization of tight junctional proteins, including Claudin-1 and Claudin-7 [12]. However, immunostaining showed that in contrast to the gradient of TROP2 expression, Claudin-7 was uniformly expressed throughout the ureteric bud cells (Figure S2A). Moreover, Claudin-7 was found only in the lateral membrane of the ureteric duct, while TROP2 was located in both the lateral and basal membranes (Figure S2B). These results indicate that expression of TROP2 and Claudin-7 are not fully correlated in the fetal kidney, suggesting that TROP2 is not only involved in the formation of tight junctions in the ureteric bud.

### The ureteric bud is surrounded by collagen-I in fetal kidney

**Figure 3:**
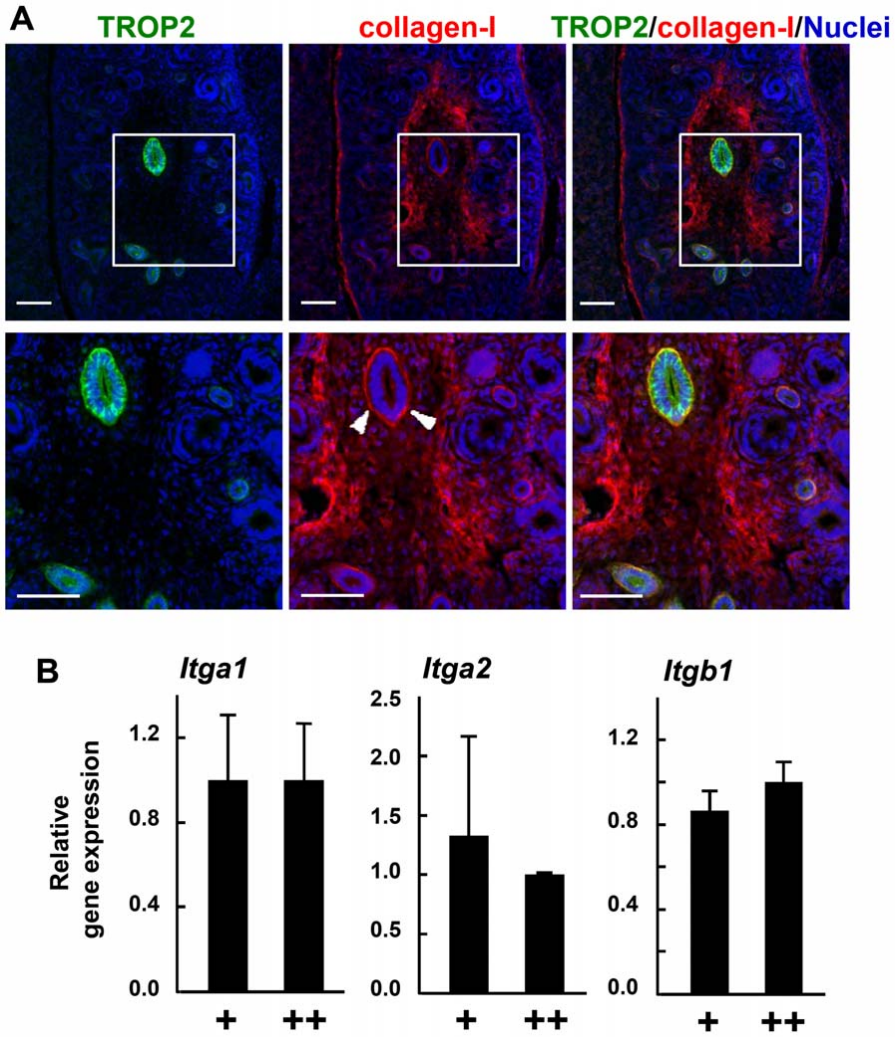
Expression of collagen-I and integrins in the fetal kidney. (A) Immunostaining of E14.5 kidney with anti-TROP2 (green) and anti-collagen-I (red) Abs and nuclei (blue). Higher magnification images of the boxed regions are shown underneath. Arrowheads indicate collagen-I surrounding the ureteric trunks. Scale bars = 100 µm. (B) Quantitative RT-PCR analysis of integrin subunits in freshly isolated EpCAM^+^TROP2^low^(+) and EpCAM^+^TROP2^high^(++) cells from E14.5 kidney. The relative gene expression of integrin/GAPDH is shown. Error bars are s.d.

The extracellular matrix (ECM) is important for the growth and branching morphogenesis of embryonic organs [29]. In fetal kidney, the ureteric bud and the collecting duct express low and high levels of collagen-binding integrins α1 and α2, respectively, suggesting collagen to play a role in the differentiation and maturation of the ureteric bud by binding to integrins [30], [31]. Because trunk cells in the ureteric bud differentiate to form the collecting duct, we examined the expression of collagen and TROP2 in the developing kidney by immunostaining. In E14.5 kidney, type I collagen (collagen-I) was abundant surrounding the TROP2^+^ trunks as well as in the mesenchyme and stroma around the ureteric bud trunk (Figure 3A). By contrast, little collagen-I was detected surrounding EpCAM^+^ tip cells (Figure S3).

Integrins are major cell-surface receptors that mediate interactions between cells and the ECM and play a crucial role in various cellular functions such as adhesion, migration, proliferation and anti-apoptosis [32], [33]. Integrins consist of α and β subunits, with the extracellular domain of the α subunit conferring their specificity. To compare the expression of collagen-binding integrins such as integrin α1β1 and integrin α2β1 between the trunk and tip cells in the ureteric bud, we performed a quantitative RT-PCR analysis of integrins using the sorted EpCAM^+^TROP2^low^ and EpCAM^+^TROP2^high^ cells from E14.5 kidney (Figure 3B). There was no significant difference in the levels of collagen-binding integrin subunits, α1, α2 and β1 between the trunk and tip cells, suggesting that the tip and trunk cells have a similar potential to bind collagen via integrins. Considering the co-localization of TROP2 and collagen-I at the basal membrane of the ureteric bud, TROP2 may play a role in the development of trunk cells such as branching by modulating the function of collagen-I.

### Tip and trunk cells exhibit different potential to attach to and spread on collagen-I

**Figure 4:**
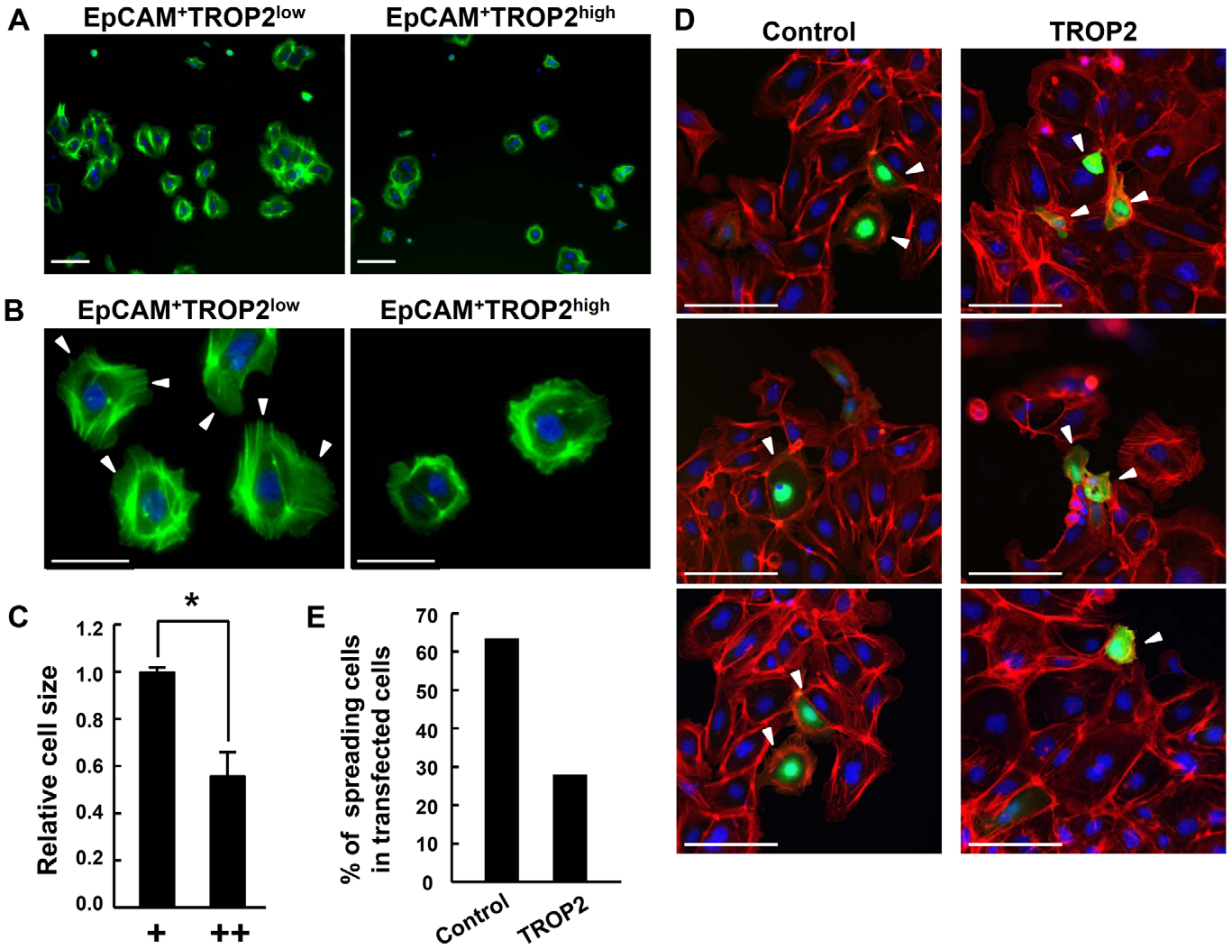
Characterization of ureteric tip and trunk cells in vitro. (A) Attachment of tip and trunk cells to collagen-coated plates. An equal number (5×10^3^ cells/well) of freshly isolated EpCAM^+^TROP2^low^ and EpCAM^+^TROP2^high^ cells from E14.5 kidney were plated on a collagen-coated 96-well plate and stained for actin filaments with Alexa488-conjugated phalloidin (green) after 24 h of culture. The fluorescence of GFP and the actin filaments was visualized under a microscope. Nuclei (blue). Scale bars = 100 µm. (B) Cell morphology of ureteric bud tip and trunk cells. Higher-magnification images of (A) are shown. Arrowheads indicate the ruffles forming at the edge of membranes. Scale bars = 50 µm. (C) Spreading of EpCAM^+^TROP2^low^(+) and EpCAM^+^TROP2^high^(++) cells. Cell size was measured using imageJ (n = 3). Error bars are s.d., * *p*<0.005. (D) Cell morphology of EpCAM^+^TROP2^low^ cells after enforced expression of TROP2. EpCAM^+^TROP2^low^ cells cultured for 24 h were transiently transfected with pTIB741 or pTIB741-TROP2 plasmid. At 24 hours after transfection, cells were stained with Alexa555-phalloidin (red). Arrowheads indicate the transfected cells expressing GFP. (E) The ratio of spreading in transfected cells. The cell of which diameter is more than two-fold of nuclear is counted as “spreading cell”. All the experiments were repeated three times and the similar results were obtained. Total number of tested transfectants is as follows: Control; 66, TROP2; 94. Representative results are shown.

To further characterize the collagen-binding properties of fetal ureteric bud cells, sorted tip and trunk cells in the ureteric buds were cultured on collagen-coated dishes. The numbers of cells attached to collagen-coated dishes were smaller for EpCAM^+^TROP2^high^ cells than EpCAM^+^TROP2^low^ cells, indicating that the trunk cells bind to collagen-I less efficiently (Figure 4A). Furthermore, while forward and side scatter by flow cytometry showed no significant difference in cell size between tip and trunk cells before plating (data not shown), the EpCAM^+^TROP2^low^ cells were spread on the collagen-coated plates and exhibited membrane ruffles, resulting in an increase in cell size compared with the EpCAM^+^TROP2^high^ trunk cells (Figure 4 B, C). It is known that new branches emerge from the tip and trunk cells form the mature ducts during the formation of ureteric bud branches, suggesting that the characteristics of tip and trunk cells in the same branch are different. These findings indicate that our primary cultures of ureteric bud cells recapitulate the differentiation process in vivo, i.e., the tip cells spread and migrate into the metanephric mesenchyme, whereas the differentiating trunk cells are less active.

Furthermore, although there was no difference in the expression of collagen-binding integrins between the tip and trunk cells in the ureteric bud, the potential of EpCAM^+^TROP2^high^ trunk cells to attach to and spread on collagen-coated plates was low, suggesting that factors other than the integrins regulate the interaction between trunk cells and collagen-I. Because TROP2 was expressed at a higher level in the trunk than tip cells and co-localized with collagen-I in the basal membrane of ureteric ducts, TROP2 may affect the potential of trunk cells to adhere to collagen-coated plates.

### TROP2 expression suppresses cell spreading

To reveal the role of TROP2 in the cell spreading on collagen-I, we sorted EpCAM^+^TROP2^low^ cells and overexpressed TROP2 by expression vector in the primary culture. Compared with the control cells transfected with the empty vector, the size of cells overexpressing TROP2 was markedly reduced, strongly suggesting that TROP2 affects the potential of EpCAM^+^TROP2^low^ tip cells to spread on collagen-I (Figure 4D, E).

To further confirm the role of TROP2 in the formation of ureteric bud branches, we utilized Madin-Darby canine kidney (MDCK) cells [34], established as a model for studying the formation of branches of ureteric bud. As no endogenous canine TROP2 was detectable in MDCK cells by RT-PCR (Figure S4A), we established MDCK cell lines stably expressing TROP2 using retroviral vectors (Figure S4B and S4C).

**Figure 5:**
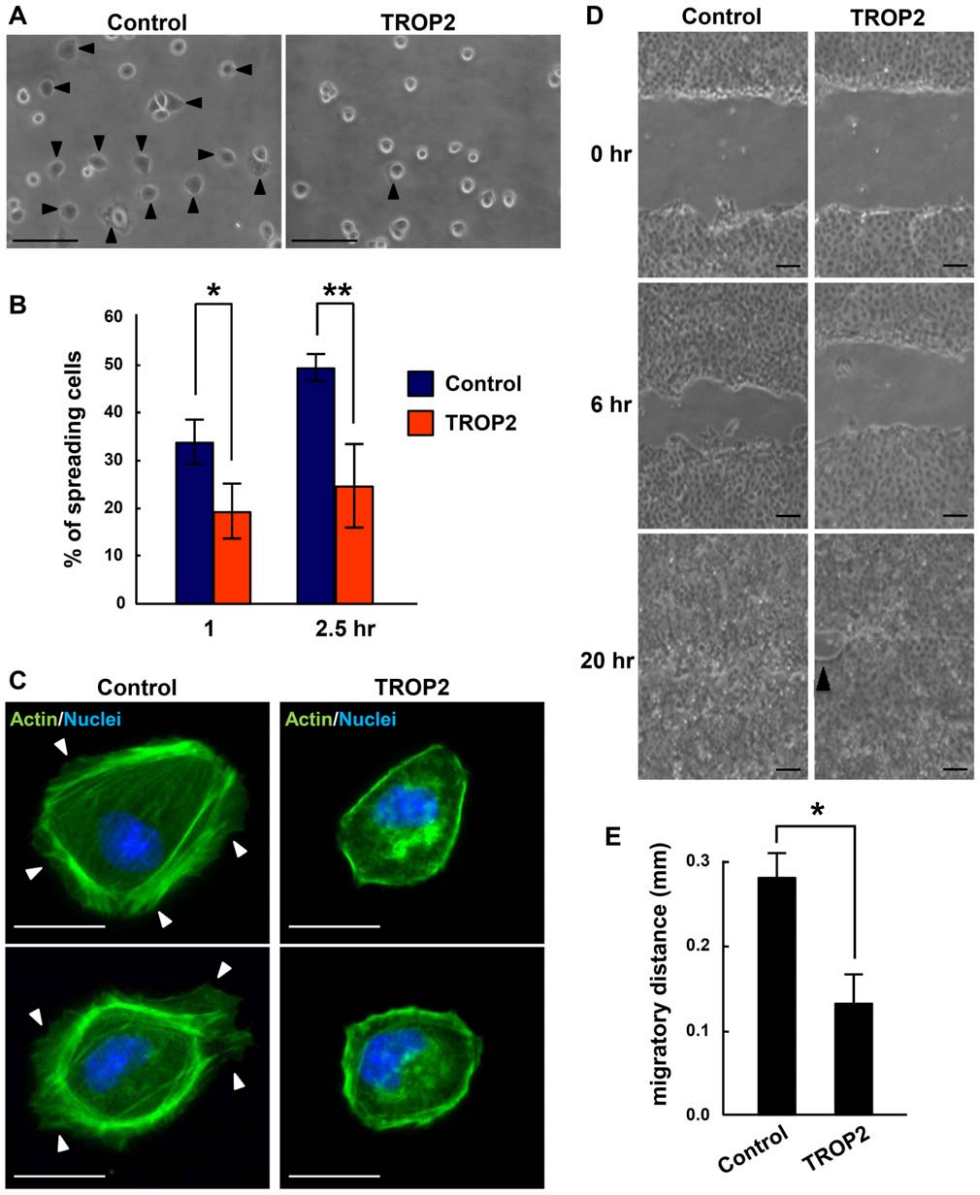
Attachment and migration of MDCK cells. (A) Cell spreading assay. Equal numbers of MDCK-control cells and MDCK-TROP2 cells, which expressed GFP and TROP2, respectively, were plated on collagen-coated glass coverslips. At 1 and 2.5 h after plating, spreading cells in several random fields were counted under a phase-contrast microscope. Arrowheads indicate spreading cells. Scale bars = 100 µm. (B) The percentage of spreading cells (n = 200). (C) MDCK-control cells and MDCK-TROP2 cells were plated on collagen-coated glass coverslips for 24 h and stained with Alexa555-Phalloidin. Arrowheads indicate the membrane ruffling at the edge of cells. Scale bars = 50 µm. (D) Wound healing assay. Cells grown as a monolayer on collagen-gel were scratched with a pipette tip. Photomicrographs were taken at 0, 6 and 16 h to record the healing process. Representative images are shown. An arrowhead indicates the unrecovered area. Scale bars = 100 µm. (E) The average migratory distance at 6 h after the scratch was measured manually (n = 3). Error bars, s.d. * *p<*0.05, ** *p*<0.01.

To confirm the results obtained with the primary cultures of ureteric bud cells, the effect of TROP2 expression on the spreading of MDCK cells was examined. Cells detached from a confluent monolayer were plated on a collagen-coated glass cover slip and images were captured at 1 and 2.5 h after plating. Control MDCK cells started to spread at least 30 min earlier than MDCK-TROP2 cells (data not shown). At 2.5 h after plating, approximately 50% of the control MDCK cells had spread (Figure 5 A, B). By striking contrast, 70% of MDCK-TROP2 cells retained a round shape without cytoplasmic extensions. This result together with the results obtained using the primary cultures indicates that the expression of TROP2 significantly suppressed spreading of kidney cells.

MDCK cells displayed membrane ruffles with cortical actin filaments and stress fibers when cultured on collagen-coated glass cover slips, whereas TROP2 expression suppressed the membrane ruffling and stress fibers (Figure 5C). Since membrane ruffling is implicated in cell motility, we examined the effect of TROP2 on cell motility with the wound healing assay. The gap in cell sheets formed by a scratch was measured at 0 and 6 h. Expression of TROP2 suppressed the migration of MDCK cells 6 h after the scratch was made (Figure 5 D, E), suggesting that TROP2 also plays a role in suppressing cell spreading and migration on collagen.

### TROP2 inhibits the branching of MDCK cells in collagen-gel culture

**Figure 6:**
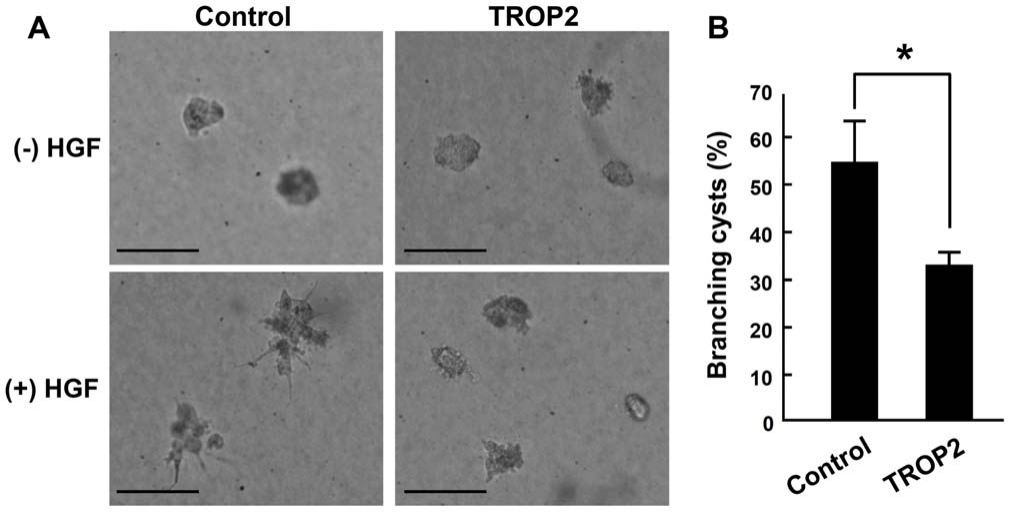
Branch formation in collagen-gel culture of MDCK cells. (A) Phase-contrast images of MDCK-control cells and MDCK-TROP2 cells cultured in 3-dimensional collagen gels. The cells were cultured without (upper panels) or with 20 ng/mL of HGF (lower panels). Both cells proliferated and formed round cysts without HGF (upper panels). Branches were formed in MDCK-control cells but suppressed in MDCK-TROP2 cells (lower panels). Scale bars = 100 µm. (B) The percentage of branching cysts in the culture with HGF. (n = 3). Error bars, s.d. * *p<*0.05.

As the spreading and migration of ureteric bud cells are necessary for the development of renal tubules, we examined the effect of TROP2 on tubulogenesis and branch morphogenesis using MDCK cells in three-dimensional collagen-gel cultures. There was no significant difference in cyst size between clusters of control MDCK and MDCK-TROP2 cells in the collagen-gel cultures (Figure 6A, the top panels). The addition of 20 ng/mL of hepatocyte growth factor (HGF), a cell scattering factor, induced morphological changes in control MDCK cells, forming branches (Figure 6A, the left bottom panel). By contrast, MDCK-TROP2 cells formed round cysts without branching (Figure 6A, the right bottom panel) and ectopic TROP2 significantly suppressed the branching formation (Figure 6B). These results suggest that TROP2 antagonizes the cell scattering activity of HGF during branch formation. These results together reveal that TROP2 suppresses cell spreading and migration on collagen-I, suggesting that it contributes to the complicated branching structures of the kidney by suppressing the formation of branches from the ureteric bud trunks.

## Discussion

TROP2 and EpCAM are structurally related molecules, however, their expression profiles differ significantly. In the E14.5 fetus, EpCAM is widely expressed in various epithelial tissues, whereas TROP2 expression is restricted to some epithelial cells in the kidney, lung and epidermis (Figure 1). During the branching morphogenesis of ureteric buds, new branches emerge from the tip through the proliferation and migration of tip cells. Meanwhile, the trunk elongates to form a rigid structure like a stalk without branching. TROP2 expression shows a gradient, increasing from the tip to trunk, suggesting that TROP2 contributes to the trunk structure (Figure 2).

ECM components play fundamental roles in the branching morphogenesis of the ureteric bud, exerting their functions through ECM-specific integrin receptors. Mice deficient in α3-integrin, a laminin-binding integrin subunit, fail to form branches of ureteric buds from the Wolffian duct, indicating laminin to be an important ECM component for branching morphogenesis [16]. On the other hand, the function of collagen-I in fetal kidney remains unknown, because mice deficient in either α1 or α2 collagen-I-binding integrin subunit do not show severe defects in the branching of ureteric buds [35], [36]. However, collagen-gel cultures of MDCK cells show that integrin α1 and α2 are essential for generating new branches and tubular structures [30], [37]. We have demonstrated here that collagen-I is present around the ureteric bud and there is a gradient of its expression from the tip to trunk during branching (Figure 3 and Figure S3). These results together suggest that collagen-I plays an important role in branching morphogenesis in fetal kidney.

An important finding of this study is the restricted expression and co-localization of TROP2 and collagen-I at the trunk of the ureteric duct. In contrast to collagen-I, where expression is restricted to that region, laminin is expressed almost uniformly around the ureteric bud (Figure S5). These expression patterns suggest a functional link between TROP2 and collagen-I. In fact, TROP2^high^ trunk cells showed reduced adhesion and spreading on collagen-I-coated plates compared to TROP2^low^ tip cells, though levels of collagen-binding integrin receptors α1, α2 and β1 were comparable between the trunks and tips (Figure 3B). To reveal the role in TROP2 for the binding and spreading of ureteric bud cells on collagen-I, we overexpressed TROP2 in TROP2^low^ tip cells and found the binding and spreading to be reduced. We also demonstrated that TROP2 expression inhibits the spreading and migration of MDCK cells on collagen-I.

Branching morphogenesis is regulated by not only the ECM but also growth factor signaling. In collagen-gel cultures of MDCK cells, a well-established model of branch formation, growth factors such as HGF and GDNF are necessary for branching [18], [38]. Studies with fetal kidney organ cultures and knockout mice have revealed the essential roles for many growth factors in branching morphogenesis. In response to growth factors, tip cells proliferate, migrate and finally form new branches, whereas trunk cells exhibit cell polarization, elongation and anti-apoptotic activity without branching [39]–[41]. In this paper we showed that TROP2 inhibits not only the attachment of cells onto collagen-coated plates but also branching in collagen-gel cultures. These results strongly suggest that TROP2 suppresses the formation of new branches at the trunk of the ureteric bud.

Focal adhesion kinase (Fak) is phosphorylated by the activation of integrins through binding to ECM and also growth factor stimulation [42]. p-Fak promotes cell extension and migration through actin polymerization and the connection of F-actin to integrin and growth factor receptors at the edge of cells, contributing to branching. In cell spreading assays, the phosphorylation level of Fak in MDCK-TROP2 cells was declined (Figure S6A), suggesting that TROP2 may inhibit the Fak signaling pathway at the ureteric trunk to avoid extra branching. While immunostaining showed that p-FAK levels are comparable in the tip and trunk cells (Figure S6B), it may be difficult to capture the transient event in static sections.

TROP2 and EpCAM are expressed in normal epithelial tissues, while they are known as oncogene and promote cancer cell proliferation and migration. EpCAM up-regulates cell motility by binding to several membrane proteins, Claudins, CD44 and tetraspanins, and α-actinin, a linker of F-actins [8], [43]. Furthermore, in cancer cells, intracellular domain of EpCAM cleaved by TACE and presenilin-2 accelerates cell proliferation through the activation of β-catenin signaling [9]. By contrast, the molecular mechanisms of TROP2 in normal and cancer tissue are not known. Immunoprecipitation assays showed that TROP2 binds EpCAM (data not shown), suggesting the possibility that TROP2 interacts with EpCAM and regulates the cell proliferation and migration activity. In ureteric bud development, TROP2 expression shows a gradient pattern in contrast to uniform expression of EpCAM. Furthermore, the TROP2 expression declines toward tip where cells are actively proliferating and migrating, suggesting that TROP2 is involved in regulation of cell proliferation, possibly via interaction with other proteins like EpCAM. Thus, the difference in expression profiles of TROP2-interacting proteins between normal and cancer cells may modulate the function of TROP2. The precise role of the TROP2 in normal and cancer tissue needs further investigation.

In the lung, which also has a tree-like structure, branching morphogenesis is induced by the interaction between mesenchyme and epithelial cells, similar to the ureteric bud [44]. However, TROP2 is not expressed in the lung (Figure 1A) and the branching pattern is clearly different from the ureteric buds in the early stages. Lung epithelial cells generate several lateral branches from trunks. By contrast, the ureteric bud generates two epithelial tips after the first branching and repeats this dichotomous branching process, and trunks form a rigid duct structure by establishing tight cell-cell junctions and suppressing the formation of new branches [45]. Specific expression of TROP2 in the kidney may result in the difference in branching patterns between the lung and kidney.

In conclusion, this study revealed a unique pattern of TROP2 expression in the ureteric bud, i.e., the expression was strongest in the trunk and gradually declined toward the tip. TROP2 and collagen-I were co-localized to the basal membrane of the trunk cells and TROP2 suppressed cell spreading and migration on collagen-I. Based on these findings, we propose that TROP2 plays a role in the morphogenesis of the ureteric bud by inhibiting unnecessary branching from the trunk. Further studies on the function of TROP2 forming a specific pattern of tubular structures should shed light on the molecular basis of branching in various organs.

## Materials and Methods

### Ethics Statement

The mice were maintained and mated in the institutional animal facility according to the guidelines of the University of Tokyo. The experimental procedures in this study were approved by the Animal Research Committee of the Institute of Molecular and Cellular Biosciences, the University of Tokyo (approval number is 23003).

### Immunohistochemistry

Mouse embryonic kidneys were fixed for 16 h at 4°C in Zamboni's solution [46], which was replaced with a 10, 15 and finally 20% sucrose solution at 4°C for 12 h, respectively. They were embedded in OCT compound and frozen. Thin sections were prepared with a cryostat (Leica). Cultured ureteric bud cells and MDCK cells were fixed in 4% PFA for 10min. Samples were incubated with the primary antibodies listed in Table S1. Signals were visualized with Alexa Fluor-conjugated secondary antibodies (Molecular Probes) used at a dilution of 1∶500. F-actin bundles were detected with Alexa Fluor 488- or 555-conjugated phalloidin (Molecular Probes) at a dilution of 1∶250. Nuclei were counterstained with Hoechst 33342 (Sigma).

### Flow cytometric analysis and cell sorting

Kidneys isolated from embryos were incubated with 0.25% trypsin and 0.5mM EDTA at 37°C for 30min and dissociated into a single-cell suspension. Cells were co-stained with fluorescein- and biotin-conjugated antibodies, washed, incubated with allophycocyanin-conjugated streptavidin (Invitrogen), and analyzed by FACSCalibur (Becton Dickinson). Dead cells were excluded by propidium iodide staining. For cell sorting, MoFlo XDP (Beckman Coulter) was used.

### RNA extraction and quantitative reverse transcription PCR (RT-PCR)

Total RNA was extracted from each cell preparation using a First Pure RNA kit (Takara). Total RNA (0.3 µg) and random hexamer primers were used to synthesize cDNA with a High Capacity cDNA Reverse Transcription Kit (Applied Biosystems). Quantitative Real-time RT-PCR experiments were conducted with a LightCycler (Roche Diagnostics) and SYBR Premix Ex Taq (Takara-bio). Primers for Glyceraldehyde-3-phosphate dehydrogenase (Gapdh) were used as the control. The pairs of primers used were as follows: 5′-TGAACGGGAAGTCACTGG-3′ (*Gapdh* primer, sense), 5′-TCCACCACCCTGTTGCTGTA-3′ (*Gapdh* primer, antisense), 5′-CTGACCTAGACTCCGAGCTG-3′ (*TROP2* primer, sense), 5′-CGGCCCATGAACAGTGACTC-3′ (*TROP2* primer, antisense), 5′-AGGGGCGATCCAGAACAACG-3′ (*EpCAM* primer, sense), 5′-ATGGTCGTAGGGGCTTTCT-3′ (*EpCAM* primer, antisense), 5′-AAGTACAGCACCAAGTTCCTCAGC-3′ (*Wnt9b* primer, sense), 5′-GAACAGCACAGGAGCCTGACAC-3′ (*Wnt9b* primer, antisense), 5′-CTGAATCAGACGCAACACTGTAAAC-3′ (*Wnt11* primer, sense), 5′-CTCTCTCCAGGTCAAGCAGGTAG-3′ (*Wnt11* primer, antisense), 5′-GGAAGGTGTCGTTGATGAAGGA-3′ (*Ret* primer, sense), 5′-CTCAGCATCCGCAATGGTG-3′ (*Ret* primer, antisense), 5′-CACTCCTGGATTTGCTGATGT-3′ (*GFR*α primer, sense), 5′-CTGAAGTTGGTTTCCTTGCCC-3′ (*GFR*α primer, antisense), 5′-CCCGATGACCAAATGAAAGACG-3′ (*Sall1* primer, sense), 5′-TAGAGAGGTTGTGATCGCTGA-3′ *(Sall1* primer, antisense).

### Culture of ureteric bud cells and transfections

Ureteric bud cells from E14.5 kidneys were suspended in DMEM/F12 (1∶1) medium (Gibco) containing 10% FBS (JRH) and antibiotics. Cells were seeded on type-I collagen-coated dishes. Relative cell size was measured as the actin-positive area using ImageJ (Rasband, W.S., NIH) and cellular area was determined. To express mouse TROP2, its cDNA fragment was inserted upstream of IRES-GFP in the vector pTIB731, provided by Dr. Tohru Itoh (University of Tokyo). Ureteric bud cells were sorted and cultured for 24 h prior to transfection. The transfection was carried out using polyethyleneimine “Max” reagent (Polysciences Inc.). Twenty four hours later, the cells were immunostained and the size of the GFP^+^ transfected cells was examined under a fluorescence microscope.

### MDCK cell culture and virus infection

MDCK cells were cultured in DMEM (Gibco) containing 10% FBS (Equitech) and antibiotics [34]. For expression of mouse TROP2, its cDNA was inserted into the pMxs vector. The pMxs-IRES-GFP vector was used as a negative control. Retrovirus was produced using the retrovirus packaging cell line PLAT-A and MDCK cells were infected as described previously [47]. Cells expressing TROP2 or GFP were sorted using anti-TROP2 Abs or GFP and MDCK cells expressing GFP (MDCK-control) or TROP2 (MDCK-TROP2) were established.

### Immunoblotting

MDCK cells were lysed in TNE buffer (20 mM Tris-HCl, pH 7.4, 150 mM NaCl, 2 mM EDTA, 1% Nonidet P-40, 1 mM phenylmethylsulfonyl fluoride, 1 mM Pefabloc SC (Roche Applied Science), and 10 µg/ml leupeptin), and the whole cell extracts were separated by SDS-PAGE and transferred onto polyvinylidene difluoride membranes (Immobilon-P; Millipore). The membranes were subjected to Western blot analyses with anti-TROP2, anti-Fak or anti-p-Fak (Tyr397) antibody. Anti-β-actin antibody was used to confirm equal loadings.

### Cell spreading assay

MDCK cells were detached by trypsin and resuspended in serum-free medium at 5×10^3^ cells/ml. The cell suspension (1 ml) was added to type I collagen-coated coverslips. Cells were allowed to spread, and spread cells were scored manually under a phase-contrast microscope at 1 and 2.5 h after plating. Spread cells were defined as the cells with extended processes.

### Wound healing assay

MDCK cells were grown on collagen-coated 60 mm dishes. The monolayer of cells was scratched with a pipette tip and the culture medium was changed to serum-free medium. Pictures were taken at 0, 6 and 16 h and the length of the gap between cell sheets was measured at 0 and 6 h.

### Collagen gel culture

MDCK cells were detached from plates with trypsin and triturated into a single cell suspension. Cells were diluted to 0.5×10^3^ cells/mL in a type I collagen solution and the cell solution was plated in a 24-well plate (500 µL/well). After incubation at 37°C to allow the collagen solution to gel, 1 mL of the culture medium was added. The medium was changed every 24 h, and after 2 days, medium containing 20 ng/mL of HGF was added and cultured for additional 2 days.

## Supporting Information

Figure S1 **Expression of TROP2 and Ki67 in E14.5 kidney.**
(TIF)

Figure S2 **Expression of Claudin7 and TROP2 in E14.5 kidney.**
(TIF)

Figure S3 **Expression of collagen-I and EpCAM in E14.5 kidney.**
(TIF)

Figure S4 **Establishment of MDCK cells expressing TROP2.**
(TIF)

Figure S5 **Expression of laminin in E14.5 kidney.**
(TIF)

Figure S6 **Phosphorylated-FAK in the tip and trunk of the ureteric bud.**
(TIF)

Table S1 **Primary antibodies used in immunofluorescence chemistry and flow cytometric analysis.**
(TIF)
