# Supplementary figures and images for: TROP2 Expressed in the Trunk of the Ureteric Duct Regulates Branching Morphogenesis during Kidney Development
Source: PLoS One. 2011 Dec 14;6(12):e28607. doi: 10.1371/journal.pone.0028607 (PMC3237457; doi:10.1371/journal.pone.0028607)

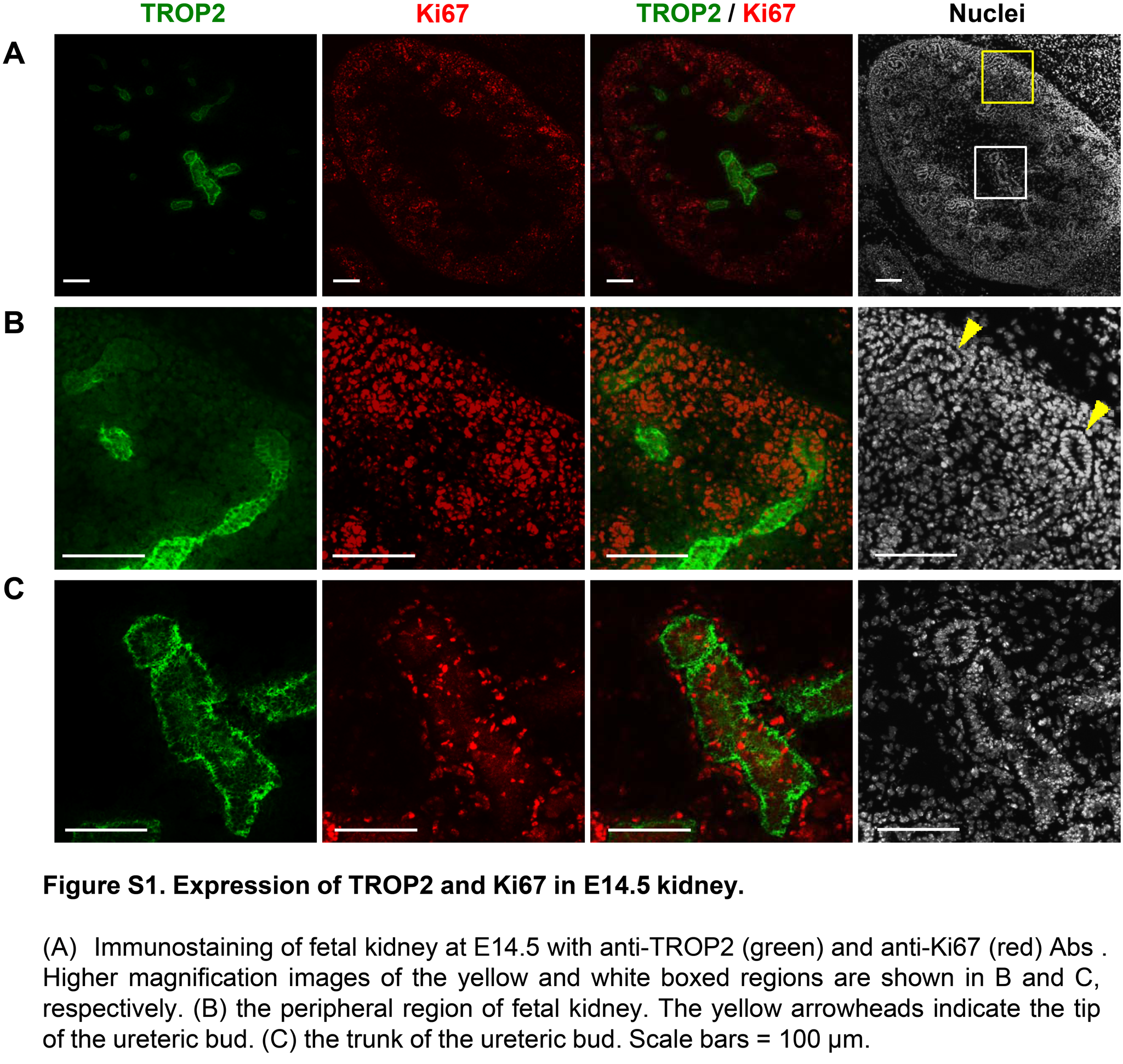

Supplement: Figure S1 — Expression of TROP2 and Ki67 in E14.5 kidney. (TIF) [file pone.0028607.s001.tif]

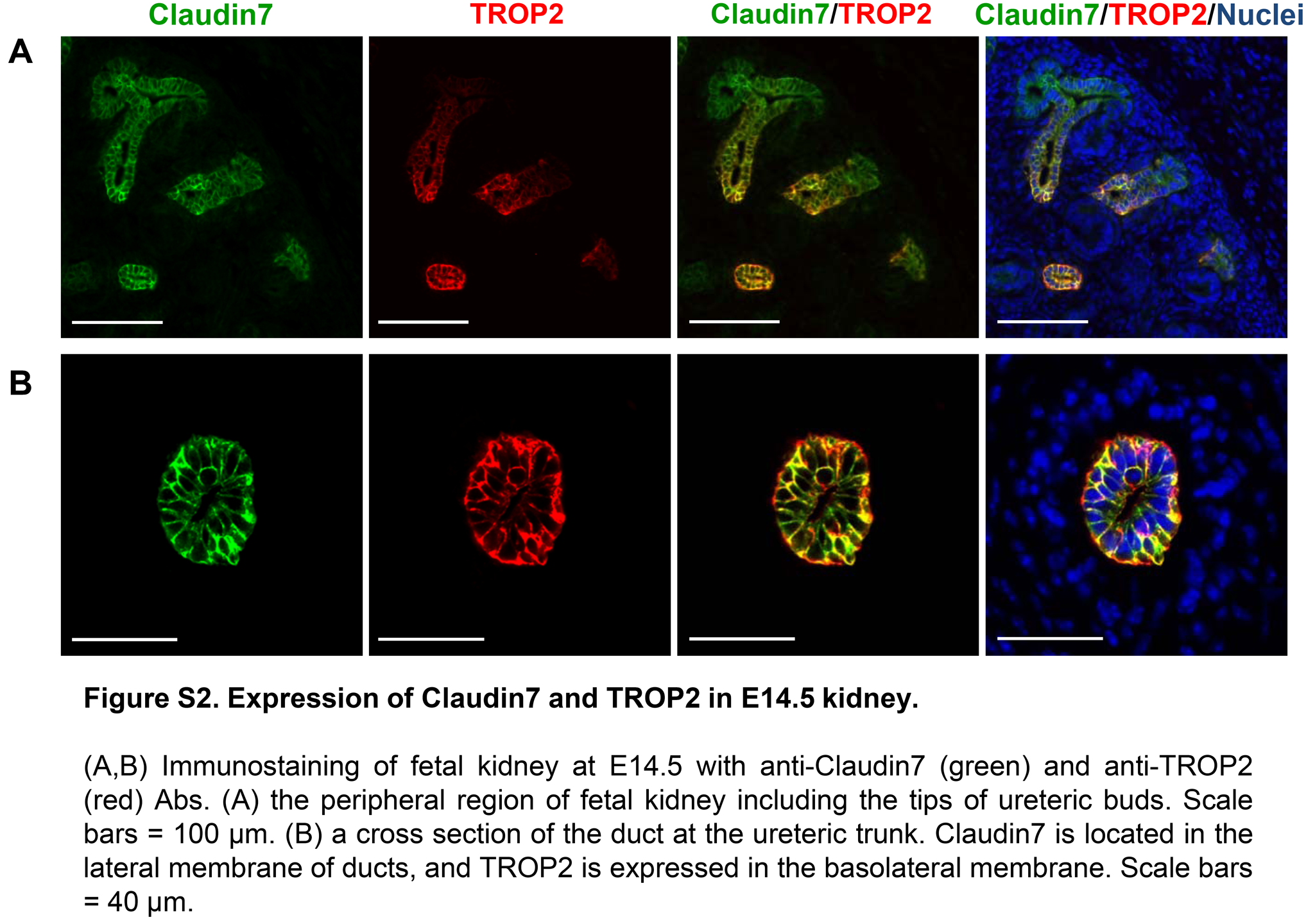

Supplement: Figure S2 — Expression of Claudin7 and TROP2 in E14.5 kidney. (TIF) [file pone.0028607.s002.tif]

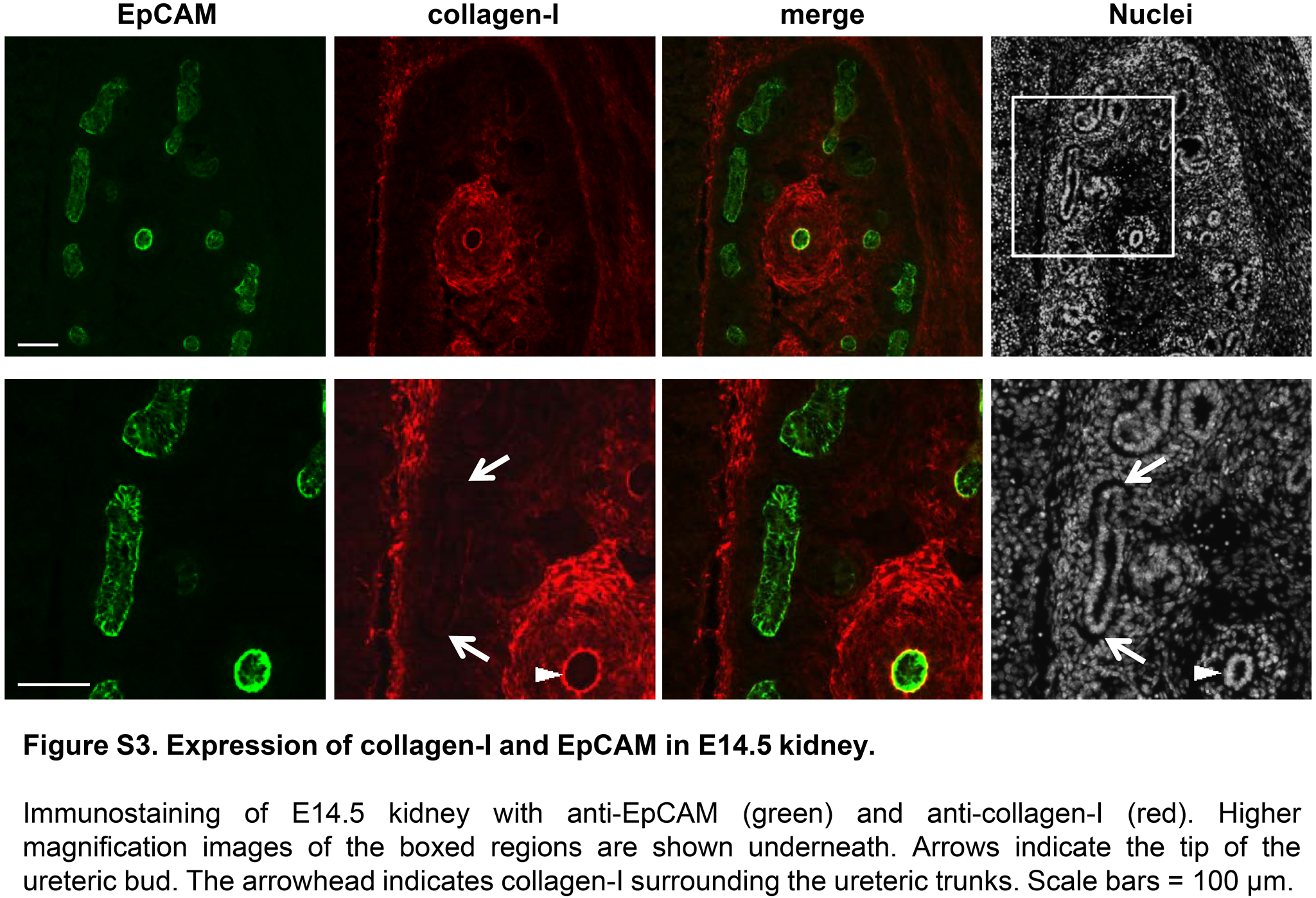

Supplement: Figure S3 — Expression of collagen-I and EpCAM in E14.5 kidney. (TIF) [file pone.0028607.s003.tif]

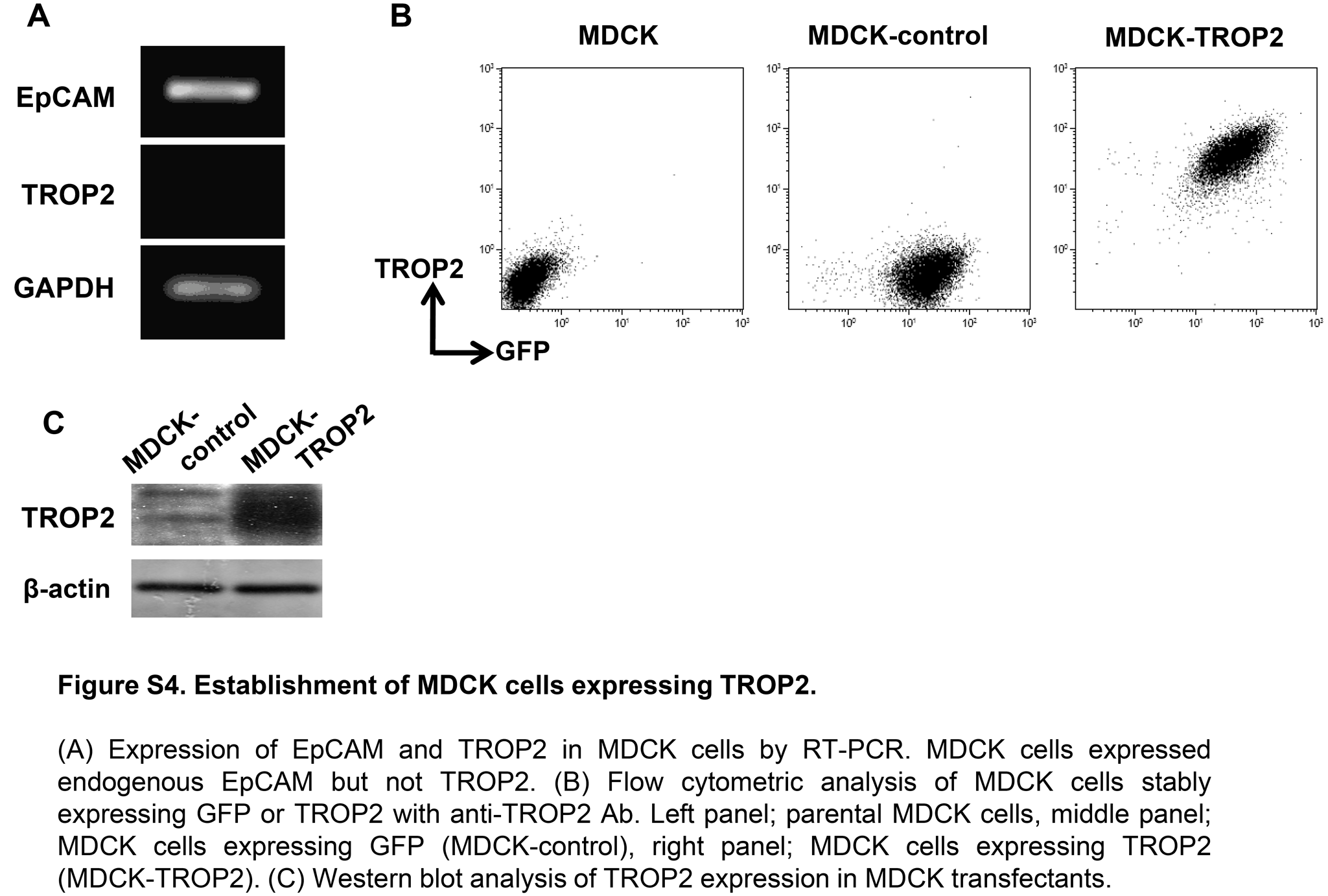

Supplement: Figure S4 — Establishment of MDCK cells expressing TROP2. (TIF) [file pone.0028607.s004.tif]

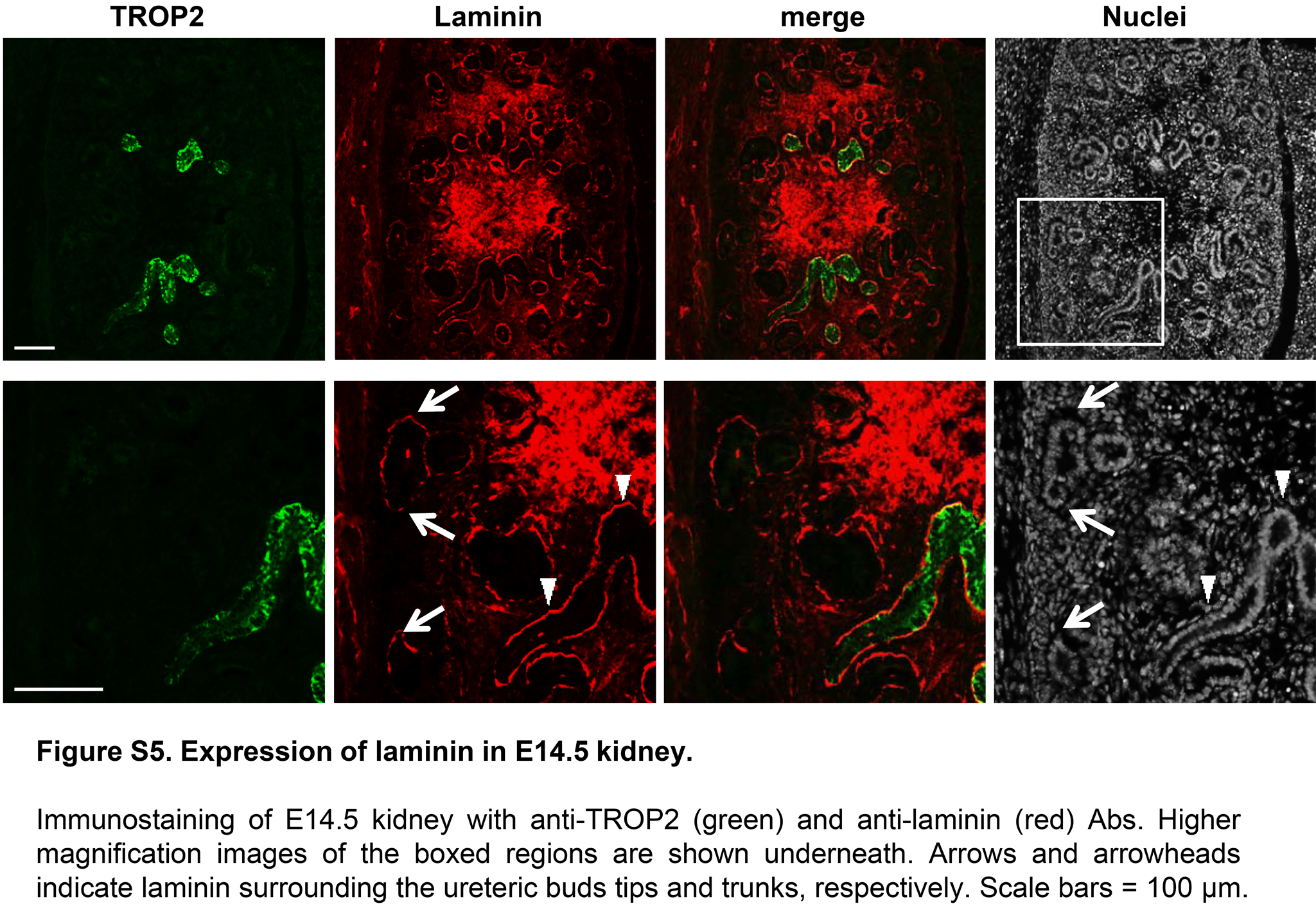

Supplement: Figure S5 — Expression of laminin in E14.5 kidney. (TIF) [file pone.0028607.s005.tif]

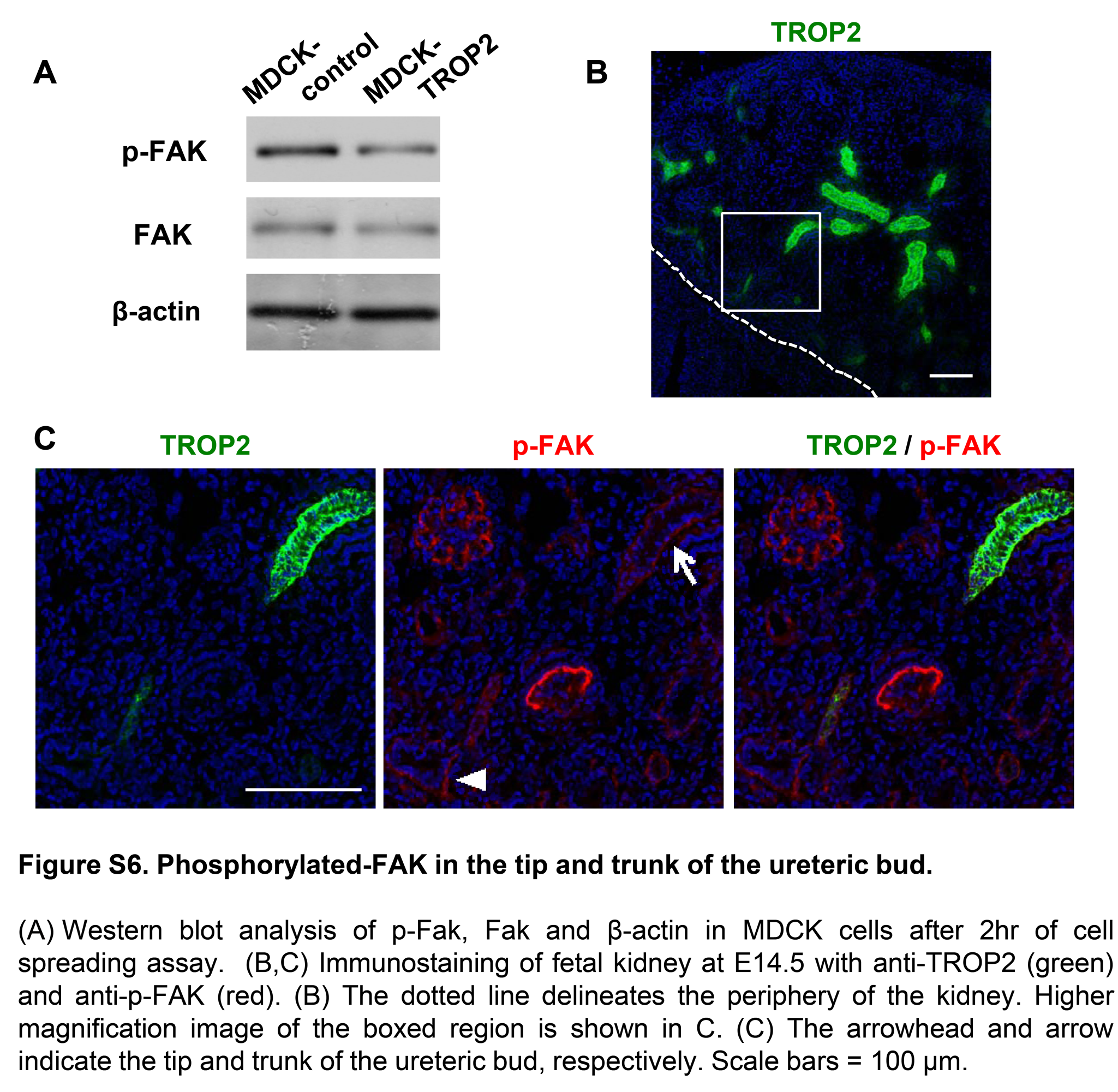

Supplement: Figure S6 — Phosphorylated-FAK in the tip and trunk of the ureteric bud. (TIF) [file pone.0028607.s006.tif]

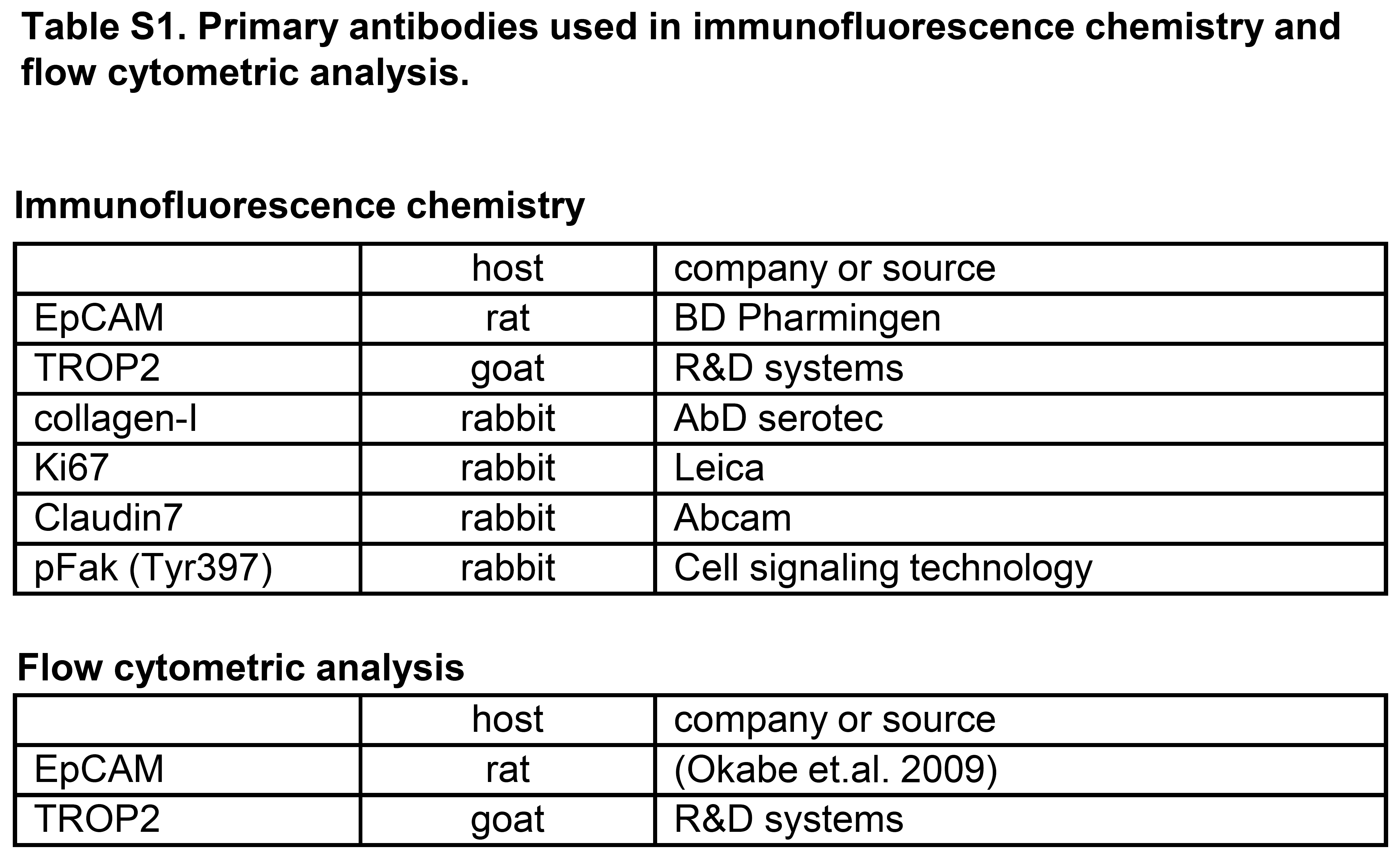

Supplement: Table S1 — Primary antibodies used in immunofluorescence chemistry and flow cytometric analysis. (TIF) [file pone.0028607.s007.tif]
